# Olanzapine for Extended-Release Injectable Suspension for Subcutaneous Use (TV-44749) Designed to Avoid the Risk of PDSS: In Vitro Release Studies in Human Plasma and In Vivo Impact of Extrinsic Factors on Pharmacokinetics

**DOI:** 10.3390/pharmaceutics18050601

**Published:** 2026-05-14

**Authors:** David Bibi, Lilach Steiner, Iva Krtalic, Marina Juretic, Biserka Cetina-Cizmek, Andrea Komlosi, Pippa Loupe, Kristina Ferderber, Hussein Hallak

**Affiliations:** 1 Innovative Research and Development, Teva Pharmaceutical Industries Ltd., Netanya 4250400, Israel; 2 Research and Development, PLIVA Croatia Ltd., Teva, 10000 Zagreb, Croatia; 3 Faculty of Pharmacy and Biochemistry, University of Zagreb, Ante Kovačića 1, 10000 Zagreb, Croatia; 4 R&D Bioanalytical Laboratory, Teva Pharmaceutical Works P. Ltd., Co. (TPW), 4042 Debrecen, Hungary

**Keywords:** olanzapine, long-acting injectable, TV-44749, schizophrenia, post-injection, delirium/sedation syndrome (PDSS)

## Abstract

**Background**: TV-44749 is a subcutaneous (sc) long-acting injectable (LAI) formulation of olanzapine that recently demonstrated efficacy and safety as a treatment for schizophrenia in adults without the occurrence of post-injection delirium/sedation syndrome (PDSS) in the phase 3 SOLARIS trial (NCT05693935). TV-44749’s sc route of administration and formulation were designed to provide prolonged olanzapine release over a monthly dosing interval and to avoid the risk of post-injection delirium/sedation syndrome (PDSS). It was designed as a copolymer in situ-forming depot technology to provide a LAI formulation that could withstand physiological and environmental factors that could affect controlled-release kinetics. **Methods**: To evaluate the robustness of the TV-44749 formulation, an in vitro release (IVR) study in human plasma was conducted, comparing TV-44749 to the commercially available intramuscular (im) long-acting injection formulation of olanzapine pamoate monohydrate. In addition, in vivo studies in rats were conducted to assess the effect of injection site manipulation following TV-44749 sc injection on olanzapine release from the depot. **Results**: The IVR study showed that upon contact with human plasma, copolymers comprising TV-44749 formulation instantly precipitate and form a solid matrix that entraps olanzapine particles. This prevents an uncontrolled release of olanzapine. Additionally, in vivo rat studies found that manipulation of the injection site after TV-44749 administration, by either heating or rubbing at different time-points, resulted in no meaningful effect on overall olanzapine exposure. **Conclusions**: The presented findings support the robustness of the TV-44749 formulation in maintaining controlled-release properties, even under conditions that could otherwise compromise release performance.

## 1. Introduction

Schizophrenia is a chronic, debilitating psychiatric disorder affecting approximately 1% of the general adult population and has higher prevalence in vulnerable populations. It is a leading cause of disability and frequently results in social and vocational exclusion [1,2,3,4]. Antipsychotics have been the basis of pharmacological treatment for over 60 years, and long-term antipsychotic treatment continuation in comparison to antipsychotic discontinuation has consistently been associated with lower all-cause and specific-cause mortality [5]. However, rates of nonadherence to oral antipsychotics range from 34% to 81%, with many studies reporting rates of approximately 50% [5]. Long-acting injectable (LAI) antipsychotics that can provide prolonged therapeutic concentrations over a period of weeks and months are an important alternative to oral medication and are particularly advantageous in the context of treatment adherence management [6,7,8,9,10].

Olanzapine has shown efficacy in first-episode schizophrenia, acute schizophrenia, acute exacerbations of schizophrenia, the maintenance treatment of schizophrenia, and treatment-resistant schizophrenia, and is associated with higher remission rates than other antipsychotic agents [11,12,13,14,15,16,17,18,19,20,21,22]. The only currently marketed olanzapine LAI is a long-acting intramuscular (im) injection formulation of olanzapine pamoate monohydrate (Zyprexa Relprevv, Zypadhera, originally marketed by Lilly, Indianapolis, IN, USA; currently marketed by CHEPLAPHARM, Greifswald, Germany) indicated for the treatment of schizophrenia [23]. The im LAI formulation is composed of an aqueous suspension of a practically insoluble olanzapine salt, which dissolves slowly at the injection site, providing sustained, therapeutic, systemic concentrations of olanzapine [24]. A serious adverse event of a post-injection delirium/sedation syndrome (PDSS) is associated with this im LAI, characterized by symptoms related to excessive sedation and/or delirium, suggestive of olanzapine overdose [23,24]. Thus, an enhanced safety program, a risk evaluation and mitigation strategy (REMS), is required to mitigate the risk of PDSS [14]. PDSS events have occurred in <0.1% of olanzapine im LAI injections and in approximately 2% of patients who received injections for up to 46 months [23], and are thought to be due to the inadvertent intravascular access of olanzapine pamoate monohydrate during the im injection process [24]. PDSS is thought to result from the significantly higher solubility of olanzapine pamoate monohydrate in plasma compared to the intramuscular interstitial environment, leading to rapid drug release upon vascular exposure. Additionally, exposure to the dynamic vascular environment, i.e., blood flow or agitation, may promote dispersion of olanzapine pamoate monohydrate particles, accelerate their dissolution and lead to unexpectedly high olanzapine plasma concentrations related to PDSS [24].

TV-44749 is an innovative LAI formulation of olanzapine intended for subcutaneous (sc) injection, and one of the critical aspects of TV-44749 development was to implement strategies to reduce the risk of PDSS. The first strategy was the use of the sc route of administration as subcutaneous tissue is much less vascularized compared to muscular tissue [25,26], reducing the likelihood of accidental vascular injury and/or intravascular injection. The second strategy was to reduce the likelihood of uncontrolled release of olanzapine. The formulation of TV-44749 is based on an in situ-forming depot technology [27] and consists of an olanzapine base, biodegradable copolymers and dimethyl sulfoxide (DMSO). The copolymers in the technology of TV-44749 are soluble in organic solvent, but insoluble in any aqueous environment. Therefore, even in the unlikely event of exposure of TV-44749 to plasma during sc injection, the polymers are expected to retain these in situ-forming properties and prevent uncontrolled olanzapine release. Thus, upon injection of TV-44749 into subcutaneous tissue, phase inversion occurs, during which DMSO diffuses out and water penetrates in, triggering the precipitation of copolymers and formation of a solid depot. This depot, composed of the copolymeric matrix with entrapped olanzapine particles, provides extended olanzapine release which results in therapeutic plasma concentrations over a 1-month period.

Another potential risk for accelerated drug release with any LAI formulation involves the vulnerability of the subcutaneous depot to different external stressors. Mechanical pressure, such as rubbing the injection site, could potentially disrupt depot integrity and increase the release rate. Similarly, exposure to elevated temperatures, whether from external sources (e.g., saunas, hot baths) or internal factors (e.g., fever), may enhance olanzapine release by potentially affecting drug solubility or compromising the structural integrity of the copolymer matrix.

In the SOLARIS phase 3 clinical study of patients with schizophrenia, no suspected or confirmed cases of PDSS have been observed following 3470 injections of TV-44749 [28]. Likewise, in the phase 1 study of patients with schizophrenia, which included rich pharmacokinetic sampling, there were no cases of PDSS and no evidence of dose dumping as TV-44749 reached clinically relevant plasma concentrations (≥10 ng/mL) within 1–2 days, with maximum plasma concentrations (C_max_) within 11–14 days, followed by a sustained release profile over the dosing period of 1 month [29]. Nonetheless, to further confirm that the controlled-release properties of TV-44749 are preserved even under the highly improbable scenario of exposure to plasma, an in vitro release (IVR) study in human plasma and nonclinical studies on the vulnerability of the subcutaneous depot to external stressors were conducted and are described here.

The IVR study in human plasma of TV-44749 was conducted in comparison to the marketed im LAI formulation of olanzapine pamoate monohydrate. As part of the IVR study, additional microscopy characterization of the TV-44749 depot was performed to assess its in situ-forming depot properties in plasma. To evaluate the robustness of the TV-44749 formulation and its susceptibility to extrinsic post-injection stressors that could induce dose dumping, in vivo pharmacokinetic studies were conducted in rats. The impact of injection-site manipulation (heating and mechanical rubbing) on olanzapine release following subcutaneous administration of TV-44749 was assessed. To this end, in vivo studies in male rats were conducted to first evaluate the suitability of rats as an animal model to assess the pharmacokinetic profile of TV-44749 and further, to determine whether heating and rubbing of the injection site following TV-44749 sc injection affects the release of olanzapine. Accordingly, the objectives of these studies were to evaluate whether the controlled-release properties of TV-44749 are maintained under stress-test conditions, including olanzapine plasma exposure and post-injection injection-site stressors, using in vitro and in vivo models.

## 2. Materials and Methods

### 2.1. In Vitro Studies of TV-44749 Following Exposure to Human Plasma: Stability, Solubility, IVR and Depot Characterization

To select the optimal parameters for the IVR analysis, the stability and solubility of olanzapine base (provided by PLIVA Croatia Ltd., Zagreb, Croatia 99.9% purity, CAS Number: 132539-06-1, molecular weight: 312.43 g/mol), the active pharmaceutical ingredient (API) in TV-44749 (Teva), and olanzapine pamoate monohydrate (obtained from Inke S.A. Castellbisbal, Barcelona, Spain with 99.9% purity, CAS Number: 221373-18-8, molecular weight: 718.83 g/mol), the API in Zyprexa Relprevv (Lilly) were tested in commercially available human plasma. Human blank plasma with K_2_EDTA as the anticoagulant was purchased from BioIVT, (Burgess Hill, West Sussex, RH15 9TN, UK). It was obtained with the statement from the manufacturer that sourcing is conducted in accordance with the highest ethical and regulatory standards. For all experiments, the pooled plasma was used. It comprised samples from both male and female donors representing Hispanic, Black, and Caucasian ethnicities, with ages ranging from 29 to 59 years. Pooled plasma was prepared in advance and consistently used across all experiments, thereby minimizing inter-donor variability. The reagents utilized for HPLC–MS/MS analysis included purified water (MilliQ, produced in-house), dimethyl sulfoxide (analytical grade, VWR, Radnor, PA, USA), and ammonium acetate (≥99%, LC–MS grade, VWR). Additional reagents supplied by Merck, Germany, comprised methanol (HPLC grade), acetonitrile (HPLC grade), isopropanol (gradient graded for HPLC), tetrhydrofuran (HPLC grade), and formic acid (98–100%, Emprove Essential grade).

The stability of both APIs in human plasma (20 mL) for 72 h at 37 °C in the air incubator was tested at two concentration levels corresponding to 5% and 50% olanzapine released in conducted IVR analysis. At defined time-points, samples were collected and filtrated through regenerated cellulose membrane 0.45 μm syringe filters. The solubility of both APIs in human plasma was determined by shake-flask method. The analysis was performed in triplicate. Each of the tested APIs was added in excess to the flasks containing 20 mL of human plasma thermostated at 37 °C. Flasks were sealed and placed on a thermostatic orbital shaker at 37 °C and stirred at 175 RPM for 28 h. The saturated API solutions were sampled for 28 h at defined sampling points to determine the equilibrium solubility. The collected samples were filtrated through regenerated cellulose membrane 0.45 μm syringe filters. After determining stability and saturation solubility, IVR analysis was conducted.

The IVR analysis in human plasma was conducted within a 0-to-72 h timeframe, which aligns with the timeframe reported in the literature for the identification and recovery of PDSS in patients [30]. For the IVR analysis, 150 mL of human plasma was placed in well closed Erlenmeyer flasks on an orbital shaking air bath. Temperature was maintained at 37 °C, and rotation was set at 100 RPM throughout the test. Six vials of both TV-44749 and Zyprexa Relprevv were reconstituted as per instructions for use and a dose of 30 mg was injected directly into the human plasma. The pH of blank human plasma prior to the IVR experiment was 7.9. At specified time-points (0.5, 1, 2, 3, 4, 6, 24, 48 and 72 h), an aliquot of sample solution was withdrawn from the Erlenmeyer flasks and immediately filtered through regenerated cellulose membrane 0.45 μm syringe filters. The plasma samples were stored frozen at −70 °C until quantification. The sampled volume was carefully replaced with 5 mL of fresh, thermostated plasma to maintain a constant total volume of 150 mL in the flask.

For the IVR analysis, a small fraction of the TV-44749 dose (30 mg, representing one-tenth of the lowest therapeutic dose) was selected, based on evidence that only a limited portion of an intramuscular suspension is likely to enter the vasculature during an inadvertent intravascular injection [24]. McDonnell and colleagues concluded that complete intravascular delivery of the full dose is highly improbable due to factors such as needle gauge, injection technique, the physical characteristics of the olanzapine pamoate suspension, and supporting pharmacokinetic data. Moreover, the substantially lower vascular density and absence of large-caliber blood vessels in sc tissue [25,26] further minimizes the probability that a clinically meaningful fraction of the TV-44749 dose could enter circulation during sc administration. Therefore, evaluating only a small fraction of the dose in the IVR study more accurately reflects a realistic accidental intravascular exposure scenario than testing the full therapeutic dose.

In addition, the selection of the 30 mg olanzapine dose was guided by the technical requirements of the in vitro release and characterization methods. Specifically, the volume to be injected for the 30 mg dose represents the volume that would reliably form a depot of uniform shape and could be consistently handled during electron microscopy characterization. Furthermore, the main premise in the conducted in vitro studies was to compare TV-44749 with Zyprexa Relprevv, using the same dose in order to assess the difference in their release properties in plasma attributable solely to the underlying drug product technologies. Finally, due to the inherent design of the IVR experiment, specifically the use of sink conditions that ensure drug release is not constrained by solubility, the obtained release profiles and conclusions are expected to be applicable across any tested dose.

Olanzapine was quantified in samples from stability, solubility and IVR testing by LC–MS/MS with an ESI ion source. Plasma concentrations were measured using a qualified LC–MS/MS method at a calibration range of 6–750 μg/mL for the quantification of olanzapine. The chromatographic system consisted of Shimadzu Prominence HPLC with a binary pump autosampler and thermostated column compartment (Shimadzu Corporation, Koyoto, Japan). A Phenomenex Synergi Hydro RP column (4 µm, 100 × 2.1 mm, Phenomenex, Torrance, CA, USA) operating at 60 °C was used to achieve good retention of olanzapine. The mobile phase consisted of 0.1% formic acid in 5 mM ammonium acetate solution (A) and 0.1% formic acid in acetonitrile (B). The initial condition was 5% B, and it was increased to 90% in 3.3 min. This composition was held for 1.1 min before returning to 5% B. The total time of analysis was 6 min to ensure re-equilibration between injections. The flow rate was 0.7 mL/min; the retention time was 2.8 min. The detection was performed on API5000 using electrospray in positive mode (ESI+). The acquisition was made in MRM mode using transition 313.2/256.2 for olanzapine and 316.2/256.2 for its isotopic internal standard (olanzapine-D3). Sample clean-up was performed by protein precipitation with acetonitrile; then, the supernatant was diluted with acetonitrile: water in 1:5 ratio before injection. During method qualification selectivity, sensitivity, accuracy, precision, response function, linearity in the required range, stability (in solution, in matrix, in sample extracts), effect of dilution, batch-size and carry-over were tested. The method proved to be selective, linear, accurate, and precise over the range of 6–750 μg/mL using 1/concentration^2^ weighting. Intra- and inter-day precision and accuracy were determined at four concentration levels in plasma samples (6, 18, 300 and 600 μg/mL). Intra- and inter-day precision (expressed in coefficient of variation) was not more than 4%. Intra- and inter-day accuracy, evaluated by using the bias between the determined concentration against the expected concentration, was between –8% and +2%. The analyte was found to be stable in human blank plasma with K2EDTA as the anticoagulant for at least 49 days at −70 °C; for 4 h at ambient temperature (bench-top stability); for three freeze–thaw cycles from −70 °C.

The depots of TV-44749 at the beginning and end of the IVR analysis were additionally characterized. The morphology (surface and cross-section) of the depots at the beginning and end of IVR analysis was evaluated by an environmental scanning electron microscope (ESEM, FEI Company, Hillsboro, OR, USA). The olanzapine particle size in the depots at the end of IVR analysis in human plasma was characterized by scanning electron microscope (SEM, JEOL Ltd. Akishima, Tokyo, Japan).

### 2.2. In Vivo Exploratory Study in Rats to Compare the Pharmacokinetic Profile of Olanzapine Following Different Olanzapine Formulations with Different Administration Routes

To determine whether the rat is a sensitive model for formulation assessment, olanzapine was administered to three groups of male Sprague Dawley (SD) rats, each group receiving olanzapine using a different olanzapine formulation and a different route of administration, and the pharmacokinetic (PK) profiles for each treated group were compared. Group 1 (*n* = 5) received olanzapine as a single intravenous (iv) injection of 0.8 mg/kg (Zyprexa powder for solution for injection, CHEPLAPHARM), Group 2 (*n* = 5) received olanzapine as a single sc injection of 1.6 mg/kg (Zyprexa powder for solution for injection, CHEPLAPHARM), and Group 3 (*n* = 5) received olanzapine as a single sc injection of TV-44749 80 mg/kg; site of injection was not touched, palpated or pressured. The target dose of 80 mg/kg corresponds to the maximum tolerated dose (MTD) of TV-44749 in male SD rats and was selected to capture the full PK profile in this exploratory setting.

Blood samples were collected from the tail vein (serial sampling) of all animals using a serial collection scheme with the following time-points: Group 1 and Group 2: pre-dose 5, 15, and 30 min, and, then, 1, 3, 6, 12, and 24 h post-dose; for Group 3: pre-dose, 15 and 30 min, and, then, 1, 3, 8, 24, 48, 96, 168, 240, 336, 504, and 672 h post-dose. Plasma concentrations were measured using a qualified LC–MS/MS method at a calibration range of 0.5–1000 ng/mL for the quantification of olanzapine. The PK analytical methods for all three PK studies are presented below.

### 2.3. Pilot Study on the Effect of Manipulation of the Injection Site on the PK Profile of Olanzapine Following TV-44749 SC Injection

The in vivo pilot study on the effect of extrinsic factors evaluated the effect of heating and rubbing at the site of injection on olanzapine PK profile during the first 24 h post-dose, following TV-44749 single sc injection in three groups of male SD rats. Group 1 (*n* = 6) received an injection of TV-44749 without manipulation at the injection site, the second group (*n* = 6) received an injection of TV-44749 followed by rubbing at the injection site, and the third group (*n* = 6) received an injection of TV-44749 followed by a heating probe applied at the site of injection. For the rubbing condition, the site of injection was rubbed for 15 s, starting at 10 min post-injection of TV-44749, using three fingers of the palm, making a lateral back and forth movement at a steady mild pace, covering the site of injection while applying mild pressure. For the heating condition, animals were anesthetized (ketamine and xylazine) after TV-44749 injection. Heating the site of injection started 10 min post-injection of TV-44749 and was established using a heating lamp calibrated to give a steady temperature of 40–42 °C, lasting for a period of 15 min. This temperature range represents a realistic level of local heating (e.g., warm compresses or hot showers) while remaining below the threshold for tissue injury for an ethically acceptable stress condition.

All three groups were given sc injections of TV-44749 70 μL at a target dose of 80 mg/kg, MTD of TV-44749 in male SD rats. Blood samples were collected from the tail vein (serial sampling) of all animals using a serial collection scheme with the following time-points: pre-dose, 10, 15 (for Groups 1 and 2 only), 30 min and 1, 2, 4, 6 and 24 h post-dose. Olanzapine plasma concentrations were measured using a qualified LC–MS/MS method at a calibration range of 50–100,000 pg/mL for the quantification of olanzapine. PK parameters were determined using non-compartment analysis, as described in the PK analytic methods section. Specifically for the PK parameters in this study, due to a >20% deviation from the target dose of 80 mg/kg for some of the animals, the actual dose was calculated per animal and the C_max_ and AUC_0–24h_ were normalized to the actual dose per animal (C_max_D_ and AUC_0–24h_D_).

### 2.4. Pivotal Study on the Effect of Manipulation of the Injection Site at Different Time-Points on the Olanzapine PK Profile Following TV-44749 SC Injection

The in vivo pivotal study of extrinsic factors evaluated the effect of heating and rubbing at the site of injection, 30 min and 4 h post TV-44749 injection, on olanzapine PK profile during the first 72 h post-dose in male SD rats. The different injection site manipulation procedures after sc injection of TV-44749 were as follows: Group 1 (*n* = 6) control, no manipulation of injection site; Group 2 (*n* = 6), site of injection was rubbed 30 min post-dose; Group 3 (*n* = 6), site of injection was rubbed 4 h post-dose; Group 4 (*n* = 6), site of injection was heated 30 min post-dose; and Group 5 (*n* = 6), site of injection was heated 4 h post-dose.

For the rubbing condition, the site of injection was rubbed for 15 s, starting at 30 min or 4 h post-injection of TV-44749, using three fingers of the palm, making a lateral back and forth movement at a steady mild pace, covering the site of injection while applying mild pressure. For the heating condition, animals were anesthetized (ketamine and xylazine) after TV-44749 injection. Heating the site of injection started 30 min or 4 h post-injection of TV-44749 and was established using a heating lamp calibrated to give a steady temperature of 40–42 °C, lasting for a period of 15 min.

All groups were given sc injections of TV-44749 70 μL at the MTD of 80 mg/kg. As part of the safety assessments, animals were weighed and examined for any clinical or local signs prior to dosing, 1 and 12 h post-dose, twice daily on days 2–4 post-dosing and before terminal bleeding. All animals were examined for signs of erythema and/or edema at 30 min, 4, 24, 48 and 72 h post-dose. Food consumption was recorded daily. Blood samples were collected from the tail vein (serial sampling) of all animals using a serial collection scheme with the following time-points: pre-dose, 10, 30 min and 1, 2, 4, 4.5, 5.5, 12, 24, 48 and 72 h post-dose. Olanzapine plasma concentrations were measured using a validated LC–MS/MS method at a calibration range of 1–1200 ng/mL. PK parameters were determined by non-compartmental analysis as described in the PK analysis section.

### 2.5. Pharmacokinetic Analyses

Pharmacokinetics were determined non-blinded from individual plasma concentration–time profiles using noncompartmental analysis (Phoenix WinNonlin version 8.1, Certara Princeton NJ, USA). Plasma concentrations below the limit of quantitation (<0.5 ng/mL for the exploratory PK study; <0.05 ng/mL (50 pg/mL) for the pilot study on extrinsic factors; < 1.0 ng/mL for the pivotal study on extrinsic factors) were designated as “BLQ”. For the purposes of calculating mean concentrations and PK parameters, all BLQ values were treated as missing and equaled zero. As the BLQ values occurred only at the very early time-points prior to measurable systemic exposure, their handling had no impact on pharmacokinetic parameters or the comparative interpretation of the study results [31]. Several PK parameters were derived, including area, under the plasma concentration–time curve (AUC) from time 0 to the time of the last measurable drug concentration (AUC_0–t_), and area under the plasma concentration–time curve from time 0 extrapolated to infinity (AUC_0–∞_). The area under the plasma concentration-versus-time curves from time zero to the time concentration at 24 h post-injection (AUC_0–24h_) and at 72 h post-injection (AUC_0–72h_) was determined by linear trapezoidal summation. The maximum plasma concentration (C_max_) was the highest observed plasma concentration and t_max_ was the corresponding time when C_max_ was observed. The half-life (T_1/2_) was the amount of time for the plasma concentration to decrease by 50%.

## 3. Results

### 3.1. In Vitro Studies in Human Plasma

#### 3.1.1. Stability and Solubility of Olanzapine in Human Plasma

Olanzapine base, the API in TV-44749, was found to be stable in human plasma up to 72 h at 37 °C at both tested concentrations. For olanzapine pamoate monohydrate, the API in Zyprexa Relprevv, the lower tested concentration was stable in human plasma up to 24 h and the higher tested concentration was stable up to 72 h. The results indicated that the stability of both APIs would be ensured during solubility and IVR analysis. The mean equilibrium solubility of olanzapine base and olanzapine pamoate monohydrate in commercially available human plasma at 37 °C was 0.371 and 0.630 mg/mL, respectively. The results show that olanzapine base has around 1.7 times lower solubility in human plasma than olanzapine pamoate monohydrate.

#### 3.1.2. In Vitro Release and Depot Characterization Study in Human Plasma

**Figure 1:**
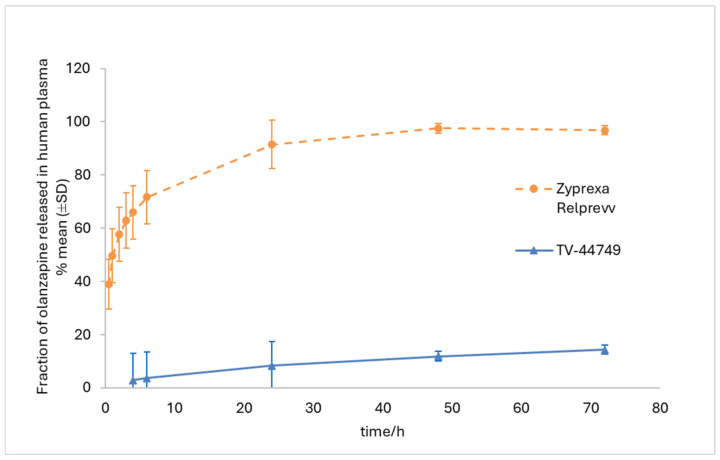
In vitro release profile of olanzapine following TV-44749 and Zyprexa Relprevv injections in human plasma at 37 °C.

The IVR results presented in Figure 1 show that Zyprexa Relprevv exhibited much faster release than TV-44749 in human plasma (calculated similarity, factor f_2_ is less than 50; f_2_ = 10). For Zyprexa Relprevv, within the first 0.5 h, a substantial fraction of the administered olanzapine dose (39%) was released, with complete release achieved within 24 h. In contrast, TV-44749 exhibited much slower and gradual olanzapine release. For TV-44749, within the first 4 h, only 3% of the administered dose was released (concentrations at earlier sampling points were below lower limit of quantification LOQ = 2%), while at the end of the IVR experiment (72 h), the release of olanzapine was far from complete: only 14% of the administered dose was released.

**Figure 2:**
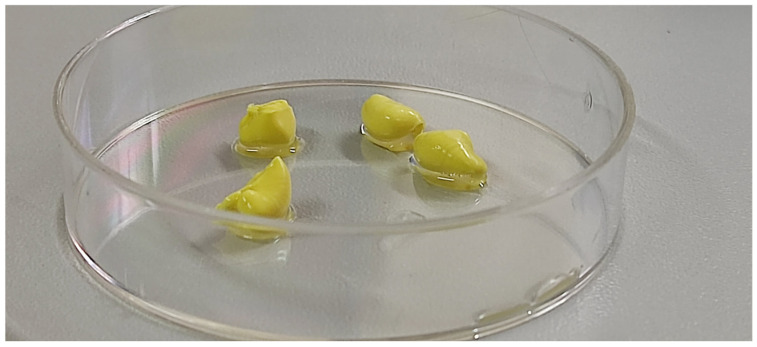
Depots of the same batch of TV-44749 suspension formed in human plasma at 37 °C in a standard Petri dish with a diameter of 90 mm.

**Figure 3:**
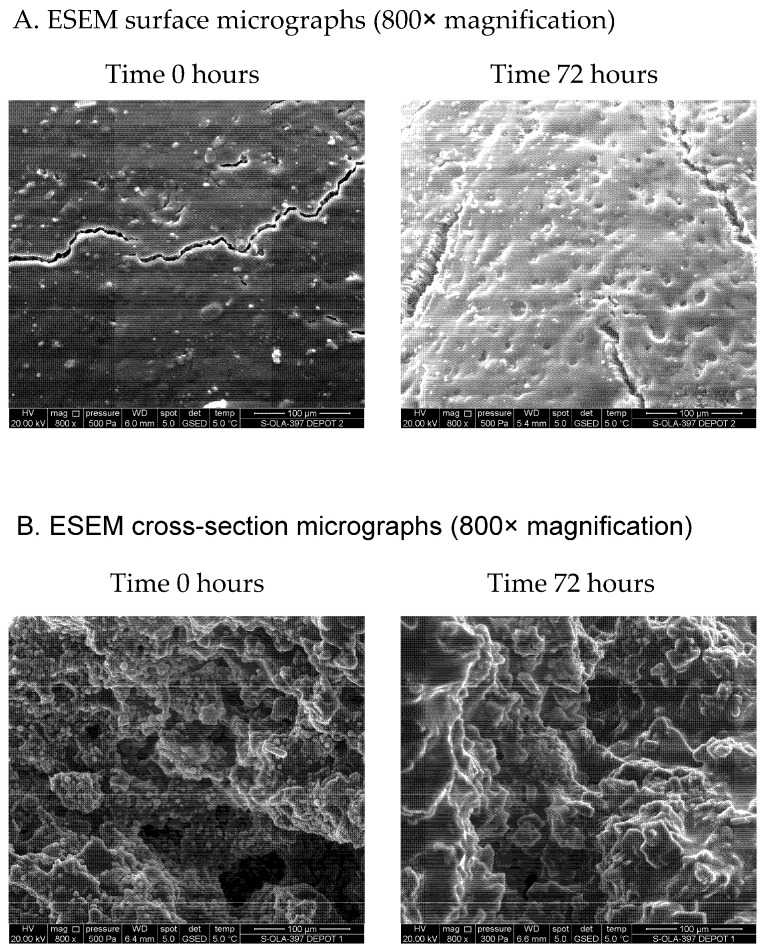
Surface and cross-section ESEM micrographs of the TV-44749 depots formed in human plasma during the IVR analysis.

Photographs of the TV-44749 depots, obtained after injecting 0.1 mL of suspension directly into human plasma (Figure 2), show that solid depots are formed instantly upon contact with plasma. Additionally, the ESEM micrographs of the morphology of both surface and cross-sections of the TV-44749 depots (Figure 3A,B) demonstrate that immediately upon exposure to plasma a well-defined surface of the depot is formed, with a solid copolymeric matrix inside entrapping olanzapine particles. At the end of the IVR analysis after 72 h, olanzapine particles are smoothly covered by copolymers, indicating that the integrity of the formed copolymer matrix was preserved after 3 days of exposure to human plasma. The observed depot morphology after 72 h in human plasma is in line with the IVR results, which show that, due to very slow olanzapine release, more than 85% of olanzapine is still entrapped within the depot. In addition to depot morphology, the particle sizes of olanzapine within the depot at the end of IVR analysis (72 h) were evaluated using SEM.

**Figure 4:**
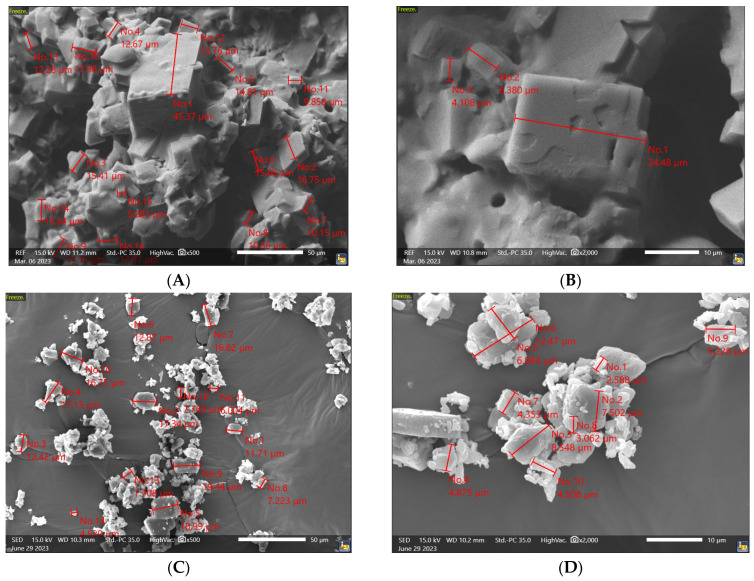
SEM images of the TV-44749 depots at 72 h: (**A**) 500× magnification and (**B**) 2000× magnification; SEM images of the TV-44749 API prior to reconstitution: (**C**) 500× magnification and (**D**) 2000× magnification.

The results of the SEM analysis, conducted on a representative 1 cm^2^ cross-sectional surface of the TV-44749 depot after 72 h exposure to human plasma (Figure 4), revealed that olanzapine particles within the depot are present as cubic crystals. The surface of olanzapine crystals contains lamellas and cracks, while the edges are smooth with some defects present, which conform to the characteristics of olanzapine base. TV-44749 is a combination drug product comprising an API containing vial and a diluent (copolymers dissolved in DMSO), supplied in a prefilled syringe intended for reconstitution before administration. Comparison of SEM images of the depots with the olanzapine API prior to reconstitution shows that the overall particle morphology is preserved during depot formation. No evidence of crystal deformation or morphological transformation was observed, indicating that the depot formation process does not alter the intrinsic crystal structure of the API. A minor difference in surface appearance was noted between the two materials, attributable to the different matrices, specifically the presence of copolymers coating the API particles within the formed depot. Semi-quantitative image analysis further demonstrated that the particle sizes of both the API prior to reconstitution and the API embedded within the depot fall within the same order of magnitude, approximately 3 to 45 µm. Overall, the SEM analysis confirms that olanzapine retains its particulate and crystalline characteristics within the TV-44749 depot, with only minor and expected differences in particle size and edge definition due to copolymer matrix interference.

### 3.2. Exploratory Comparison of Olanzapine PK Profiles Following TV-44749 SC Injection and Olanzapine Powder for Solution Administered as IV and SC Injections

The PK parameters for olanzapine following administration of TV-44749- and olanzapine-administered iv and sc injections are shown in Table 1. Following sc injections of TV-44749 80 mg/kg and olanzapine 1.6 mg/kg, low variability of exposure parameters (coefficient of variation (CV%) = 21–30) was observed compared to relatively intermediate variability of exposure parameters (CV% = 68) following the olanzapine solution 0.8 mg/kg iv dose. The absolute bioavailability of the TV-44749 sc dose and the olanzapine sc injection of 1.6 mg/kg were greater than 100%, suggesting complete absorption.

**Figure 5:**
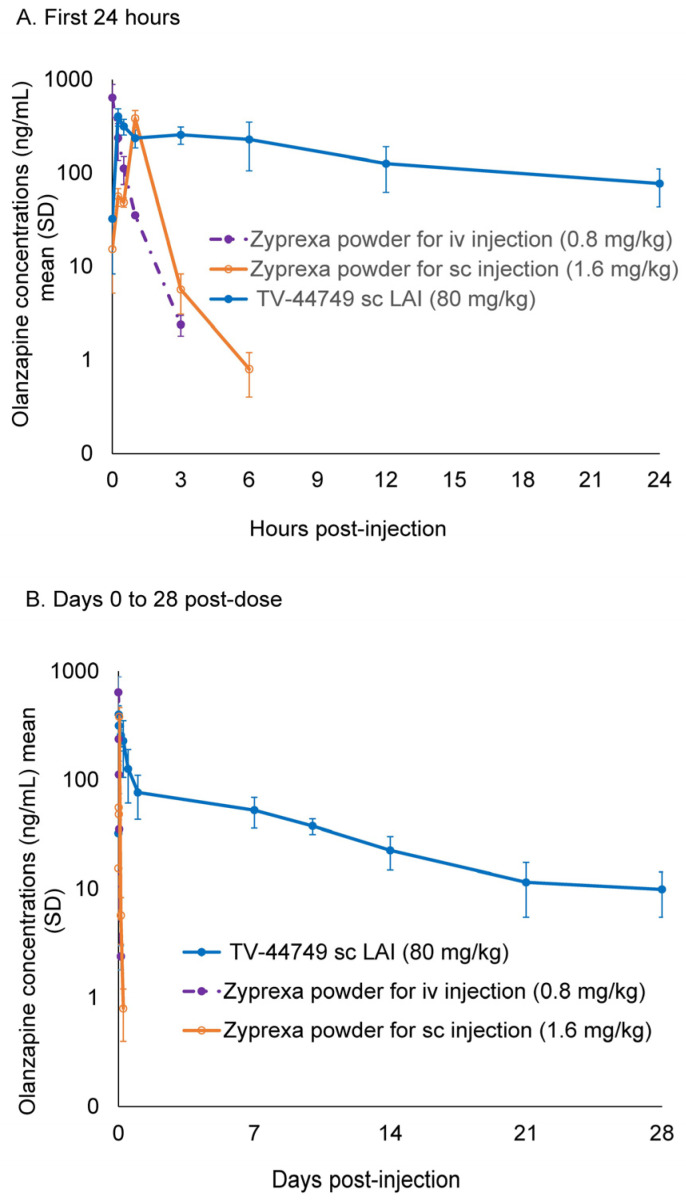
Mean (SD) PK profile of olanzapine for the different treatment groups in the exploratory study.

TV-44749 displayed an extended-release profile with an initial increase in concentration reaching C_max_ by 0.5 h, followed by a drop and second lower peak around 8–24 h, suggesting complex absorption (Figure 5A). As shown in Figure 5B, olanzapine plasma levels following TV-44749 injections gradually decreased afterward with full elimination by day 28.

### 3.3. Effect of Extrinsic Factors on Olanzapine PK Profile Following TV-44749 SC Injection in the Pilot Study

**Figure 6:**
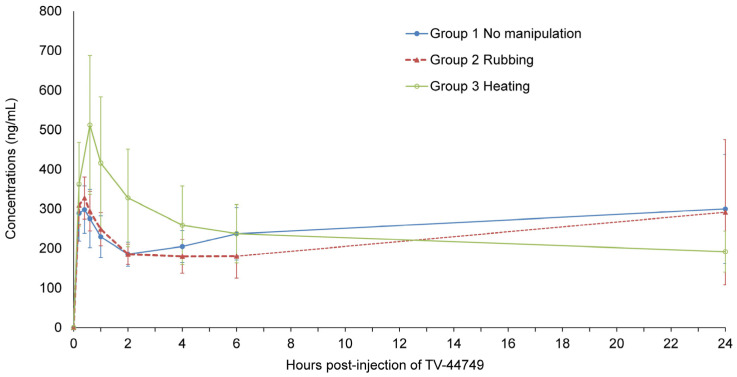
Mean (±SD) olanzapine concentrations in plasma after rubbing and heating of the injection site following single TV-44749 sc dose in the pilot study. Group 1 received no manipulation at injection site; Group 2 received rubbing for 15 s at injection site, 10 min post-dose; Group 3 received heating for 15 min at injection site, 10 min post-dose.

The pilot study on extrinsic factors evaluated the effect of rubbing and heating conditions applied to the site of injection 10 min post-dose on the PK profile, following a sc injection of TV-44749. The time to reach maximum concentration, t_max_, ranged from 15 to 30 min post-dose with a second moderate elevation in concentration from 4 to 6 h to 24 h post-dose in some of the animals, regardless of treatment group (Figure 6). Intermediate variability in exposure parameters (32–42%) was observed in all three treatment groups (Table 2). The relative exposure between Groups 1 (no manipulation of injection site) and 2 (rubbing of injection site), and between Groups 1 and 3 (heating of injection site) for C_max_D_ and AUC_0–24h_D_ were calculated as (Group 2 and Group 3 mean C_max_D_/Group 1 mean C_max_D_) × 100 and (Group 2 and Group 3 mean AUC_0–24h_D_/Group 1 mean AUC_0–24h_D_) × 100, respectively. The C_max_D_ ratios were 119% for Group 2 and 163% for Group 3; thus, rubbing the site of injection for 15 s had a low effect (19%), while heating the site of injection resulted in a 63% increase compared to the non-manipulated group. AUC_0–24h_D_ values were similar between the three groups, and the AUC_0–24h_D_ ratios were 89% for Group 2 and 100% for Group 3.

### 3.4. Effect of Heating and Rubbing the Site of Injection 30 Min and 4 H Following the TV-44749 SC Injection on the Olanzapine PK Profile in the Pivotal Study

**Figure 7:**
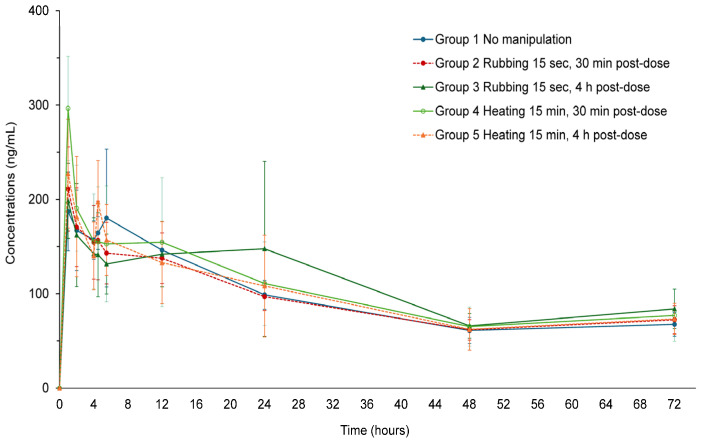
Mean (±SD) PK profile of olanzapine with or without manipulation of injection site 30 min or 4 h post-TV-44749-injection in the pivotal study.

In the pivotal study, olanzapine plasma concentrations for all groups increased steadily, reaching C_max_ 10 min to 24 h post-injection, with a median T_max_ of 12 min (0.20 h, see Table 3). In most animals, C_max_ occurred before the injection site manipulations at 30 min and 4 h post-injection. A second peak in concentration occurred between 8–12 h in most animals across all groups, and plateau concentrations lasted up to 72 h post-dose (Figure 7). Mean C_max_ values in Groups 1–5 were 232.8, 283.6, 290.0, 324.0, and 310.1 ng/mL, respectively. The relative C_max_ ratios between the groups were calculated as (Group 2 to Group 5 mean C_max_/Group 1 mean C_max_) × 100. The relative C_max_ ratios for Groups 2, 3, 4, and 5 were 121%, 124.5%, 139.2%, and 133.2%. Relative exposure (AUC_0–72h_) ratios between the treatment groups were calculated as (Group 2 to Group 5 mean AUC_0–72h_/Group 1 mean AUC_0–72h_) × 100. The AUC_0–72h_ values were similar between Groups 1–5, with the relative exposure ratios for Groups 2, 3, 4, and 5 as 98%, 111%, 104%, and 97%. The exposure from time zero to infinity (AUC_0–∞_) could not be calculated due to large extrapolations (>20%). In all five treatment groups, TV-44749 was well tolerated in rats after a single sc injection, and no significant changes were observed in body weights and food consumption. Decreased activity, which is a known effect of olanzapine, was observed in all animals in all groups up to day 3. No differences were noted in local tolerability between animals subjected to rubbing or heating at the injection site compared to animals that were not subjected to rubbing or heating. Mild adverse injection site reactions (slight erythema and edema) were observed in one animal in Group 4 and two animals in Group 5. It was also possible that the leakage of DMSO might cause undesired effects on the skin. All instances of formulation leakage or minor bleeding immediately after injection were reviewed and compared to animals showing no such findings. There were no consistent differences in local skin responses identified between these groups. One animal from Group 5 was found dead on the morning of day 2. The cause of death remained idiopathic, as olanzapine concentrations in this animal up to day 2 were similar to concentrations in the rest of the group. The animal did not show any abnormal clinical behavior on day 1 and behaved comparably to the other animals. Therefore, while the death is reported, no clear causal relationship can be established based on the available observations.

## 4. Discussion

LAI formulations using modern drug delivery systems have greatly increased antipsychotics treatment success due to increased patient adherence because of more convenient and less frequent dosing [10]. As an in situ-forming depot LAI formulation for subcutaneous administration, TV-44749 was designed to provide controlled release of olanzapine and to avoid the risk of PDSS. The recent phase 3 SOLARIS trial demonstrated the efficacy and safety of TV-44749 in patients with schizophrenia, with no suspected or confirmed PDSS events [28,32]. The IVR and depot characterization studies, together with the preclinical extrinsic stressor studies, were conducted to evaluate the potential for uncontrolled olanzapine release.

The results of the IVR study showed that TV-44749 exhibited controlled release properties in human plasma, with no indication of uncontrolled and rapid olanzapine release. The slower release of olanzapine from TV-44749 compared to Zyprexa Relprevv can be partially attributed to the 1.7-fold lower solubility in human plasma of the olanzapine base of TV-44749 relative to the olanzapine pamoate monohydrate of Zyprexa Relprevy. However, the magnitude of the observed difference in release rates between the two formulations exceeds what can be explained by solubility differences alone. This suggests that the primary factor contributing to the slower release from TV-44749 lies in the distinct formulation characteristics, particularly the in situ-forming depot technology. More precisely, for TV-44749, instantly upon contact with plasma, a clearly formed surface of the depot is observed with a solid copolymeric matrix inside entrapping olanzapine particles. This depot structure remains intact even after 3 days of continuous plasma exposure, indicating strong structural integrity and sustained release behavior. In contrast, for the aqueous suspension Zyprexa Relplevv, the release kinetics are governed by the API solubility, with no excipients controlling the drug release [24]. Therefore, in contact with plasma, olanzapine pamoate monohydrate particles are quickly dispersed within the agitated plasma volume and consequently undergo a rapid dissolution process, reflecting the conditions of the suspension exposure to bloodstream motion. As IVR studies do not fully replicate whole-blood hydrodynamics, they reliably represent the exposure of a formulation to plasma under sink conditions and can effectively differentiate between the two drug product technologies. Overall, the presented results suggest that exposure to human plasma does not compromise the controlled-release properties of TV-44749.

Another safety aspect assessed during TV-44749 development included different real-life conditions that could potentially compromise the controlled-release properties of in situ-forming depot LAIs. The results of the first exploratory study confirmed that the rat model was a sensitive and reliable model. TV-44749 demonstrated a long and durable release profile similar to that observed in humans [29], with complete elimination by day 28. Given the close resemblance between rat and human PK profiles shown for TV-44749, the rat was considered an appropriate species for exploring the impact of extrinsic factors on the controlled release properties of TV-44749. In the pilot study on the effects of injection site manipulation, heating the site of TV-44749 injection 10 min post-dose resulted in a higher C_max_ compared to control; however, the elimination phase was similar to that of the control group, with no observed differences in exposure (AUC_0–24h_), suggesting no dose dumping occurred. These observed differences in C_max_ between the control group and the group subjected to injection site heating following TV-44749 injection suggest that the rat is a sensitive and reliable test system for the evaluation of the effect of TV-44749 injection-site extrinsic factors on the pharmacokinetics of olanzapine. However, although the 80 mg/kg was selected to provide a sensitive stress-test of depot robustness, quantitative translation of high-dose rat PK effects to clinical dosing is limited; accordingly, these results are presented as nonclinical stress-testing rather than as a direct predictor of clinical exposure.

In the pivotal study, the effect of manipulation of the injection site at different times post-TV-44749-sc-injection on the PK profile of TV-44749 was examined, in which heating or rubbing was performed 30 min or 4 h post-injection with 72 h of monitoring. Overall, TV-44749 sc injections were well tolerated in all groups; decreased activity, a known side effect of olanzapine, occurred in all animals, and there were no differences due to heating or rubbing the injection site. In all groups, olanzapine plasma concentrations increased, reaching C_max_ within 10 min post-dose (first plasma collection time-point) with a second peak between 8–12 h in most of the animals, suggesting complex absorption followed by a decline to plateau concentrations up to 72 h. In most animals, C_max_ occurred before the application of the rubbing or heating conditions; therefore, any variations in C_max_ cannot be attributed to the treatment. In some animals, a moderate elevation in olanzapine plasma concentrations occurred after the treatments. However, no meaningful effects on olanzapine terminal phase or exposure were observed, as reflected by the comparable AUC_0–72h_ values across all five groups. These findings are descriptive in nature, as the limited sample size precluded robust statistical inference; therefore, no formal statistical analyses were performed. Nevertheless, the data demonstrate a consistent trend across groups.

In conclusion, the conducted in vitro and in vivo studies highlight the robustness of the TV-44749 formulation in withstanding various conditions that could potentially compromise its controlled-release properties. The IVR study demonstrates TV-44749 ability to retain in situ-forming properties under exposure to human plasma, whereby the copolymers instantly formed a depot that entraps olanzapine particles, preventing their rapid dissolution and maintaining controlled release. The PK studies in rats show that applying rubbing or heating to the TV-44749 injection site had minimal impact on overall olanzapine exposure.

## Figures and Tables

**Table 1:** Pharmacokinetic parameter values for olanzapine after different treatment groups in the exploratory study.

Mean (SD) CV%	Group 1 Zyprexa Powder iv injection 0.8 mg/kg (*n* = 5)	Group 2 Zyprexa Powder sc injection 1.6 mg/kg (*n* = 4) ^c^	Group 3 TV-44749 sc injection 80 mg/kg (*n* = 5)
C_max_ (ng/mL)	1119.7 (1115.4) 99.6% ^b^	382.8 (80.7) 21.1%	400.2 (83.1) 20.8%
AUC_0–t_ (ng × h/mL)	216.9 (179.8) 68.0%	516.9 (102.1) 19.5%	29,472.6 (9076.1) 29.7%
AUC_0–∞_ (ng × h/mL)	218.3 (179.5) 67.6%	523.1 (116.1) 22.1%	31,420.6 (8834.6) 20.8%
T_1/2_ (h)	0.3 (0.2) 44.9%	0.6 (0.05) 9.9%	127.7 (34.4) 25.2%
t_max_ (h) ^a^	-	1.0 (1.0–1.0)	0.5 (0.5–0.5)

^a^ Median (min–max range). ^b^ Reported as concentration at time zero. ^c^ Means include all dosed animals except one in Group 2 who did not meet the terminal slope criteria. SD = standard deviations; CV% = coefficient of variation.

**Table 2:** Olanzapine PK parameters in the first 24 h post-dose of a single sc injection of TV-44749 after manipulation of injection site in the pilot study.

Mean (SD) CV%	Group 1 No manipulation of injection site (*n* = 6)	Group 2 Rubbing injection site for 15 s, starting 10 min post-dose (*n* = 6)	Group 3 Heating injection site for 15 min, starting 10 min post-dose (*n* = 6)
t_max_ (h) ^a^	0.3 (0.2–24.0)	0.4 (0.2–24.0)	0.5 (0.5–1.0)
C_max_ (ng/mL)	320.8 (119.6) 35.1%	379.4 (125.8) 29.3%	492.1 (173.8) 33.2%
C_max_D_ (ng/mL)	5.9 (2.3) 39.0%	7.0 (2.5) 35.7%	9.6 (3.5) 36.5%
AUC_0–24h_ (ng × h/mL)	5842.5 (2040.7) 34.5%	5148.2 (2063.9) 38.3%	5527.6 (1599.4) 30.4%
AUC_0–24h_D_ (ng × h/mL)	106.7 (39.4) 36.9%	95.4 (39.6) 41.5%	107.5 (34.8) 32.4%

^a^ Median (min–max range).

**Table 3:** Olanzapine PK parameters after 72 h post-dose of a single sc injection of TV-44749 with or without manipulation of injection site in the pivotal study.

Mean (SD) CV%	Group 1 No manipulation of injection site (*n* = 6)	Group 2 Rubbing injection site for 15 s, starting 30 min post-dose (*n* = 6)	Group 3 Rubbing injection site for 15 s, starting 4 h post-dose (*n* = 6)	Group 4 Heating injection site for 15 min, starting 30 min post-dose (*n* = 6)	Group 5 Heating injection site for 15 min, starting 4 h post-dose (*n* = 6)
t_max_ (h) ^a^	0.2 (0.2–5.5)	0.2 (0.2–0.5)	0.2 (0.2–24)	0.2 (0.2–1.0)	0.2 (0.2–0.2)
C_max_ (ng/mL)	232.8 (57.5) 24.1%	283.7 (64.7) 21.8%	290.0 (84.1) 31.0%	324.0 (44.6) 13.7%	310.1 (64.5) 21.1%
AUC_0–72h_ (ng × h/mL)	6878.5 (1000.7) 13.6%	6718.4 (1210.4) 18.0%	7637.1 (2257.8) 30.0%	7151.4 (2644.3) 34.4%	6695.9 (2022.1) 33.6%

^a^ Median (min max range).

## Data Availability

The original contributions presented in these studies are included in the article. Further inquiries can be directed to the corresponding author.
